# How gist and association affect false memory: False recognition and gist rating norms

**DOI:** 10.3758/s13428-025-02681-8

**Published:** 2025-04-29

**Authors:** Minyu Chang, C. J. Brainerd, Daniel M. Bialer, Xinya Liu

**Affiliations:** 1 Department of Psychology, Trinity University, One Trinity Place, San Antonio, Texas 78212 USA; 2 Department of Psychology and Institute for Human Neuroscience, Cornell University, Ithaca, NY USA; 3 Department of Psychological Science, University of Arkansas, Fayetteville, AR USA

**Keywords:** False memory, DRM illusion, Normed data, Fuzzy-trace theory, Activation/monitoring framework

## Abstract

The Deese/Roediger/McDermott (DRM) illusion is one of the most widely used methods for studying false memory. Early studies provided normed false recall and false recognition data for DRM lists, where recognition is preceded by prior recall tests, and reported regression analyses that revealed backward associative strength (BAS) as one of the strongest predictors of false memory. As an extension of that line of research, we collected new recognition norms that are not confounded by prior recall tests and included gist strength (GS) as a theory-driven predictor of false memory. In Study 1, we normed true and false recognition for 55 DRM lists without prior recall tests, and in Study 2, we normed these lists for their perceived levels of gist strength. In Study 3, we fit a series of multiple linear regression models to the recognition data from Study 1 as well as recall and recognition data from prior false memory norms to disentangle the effects of BAS and GS on false recognition (with and without prior recall) and immediate false recall. Our results revealed that levels of true recognition and the recall–recognition correlation were inflated by prior recall tests. More importantly, GS was the strongest predictor of false recognition, whereas BAS was the strongest predictor of immediate false recall. A GS × BAS interaction was consistently observed for false recall and false recognition, in which the effects of BAS declined as GS increased. This suggests that the two variables compete with each other rather than reinforce each other’s effects.

## Introduction

False memory refers to the recognition or recall of events that were never experienced or were experienced differently than they are remembered. This phenomenon has been heavily researched, owing to its combination of theoretical importance and empirical implications for situations in which memory accuracy is crucial (e.g., eyewitness identification, medical histories; Brainerd & Reyna, 2005). For some years, the Deese/Roediger/McDermott illusion (DRM; Deese, 1959; Roediger & McDermott, 1995) has been one of the most widely used paradigms for studying false memories under controlled conditions. In this simple list-learning paradigm, participants encode a list of words (e.g., *nurse*, *sick*, *hospital*, *medicine*, …) that are associates of a common missing word, which is called a critical distractor (e.g., *doctor*). The standard finding is that participants often falsely remember the critical distractor as being on the list, and this effect is robust across many manipulations and populations (Gallo, 2006).

A key practical advantage of the DRM illusion is the paradigm’s remarkable adaptability. This allows researchers to tailor the procedure to investigate false memory effects across a vast array of manipulations and experimental designs. For instance, it can be easily adapted to study emotional false memories (Bookbinder & Brainerd, 2016; Budson et al., 2006; Chang et al., 2021; Otgaar et al., 2017; Zhang et al., 2017; for a review, see Wiechert et al., 2023) and phonological false memories (Finley et al., 2017; Sommers & Lewis, 1999; Watson et al., 2003; for a review, see Chang & Brainerd, 2021). Further, it can be adapted to accommodate the performance limitations of special populations, such as young children, older adults, and older adults with dementia (e.g., Abadie et al., 2021; Balota et al., 1999; Brainerd & Reyna, 2007; Holliday & Weekes, 2006; Lampinen et al., 2006), and the methodological constraints of neuroimaging research (Dennis et al., 2012; Gilmore et al., 2019; Kurkela & Dennis, 2016).

Yet another advantage of the DRM illusion is the availability of list pools that have been normed for their true and false memory levels. The most extensive example is a pool of fifty-five 15-word DRM lists that were assembled by Roediger et al. (2001). This pool is an expansion of a pool of thirty-six 15-word lists that were normed by Stadler et al. (1999). Roediger et al. compiled the list-level true and false recall as well as true and false recognition data for the 36 DRM lists from Stadler et al. and for a further 19 lists from Gallo and Roediger (2002). Overall, the list pool displayed a wide range of variability in both false recall and false recognition, which makes it an attractive standardized tool for manipulating baseline levels of false memory in experimental designs.

Notably, both Stadler et al. (1999) and Gallo and Roediger (2002) used a procedure in which recognition tests were administered following immediate recall tests for these lists. In that connection, some of the earliest DRM experiments (e.g., Payne et al., 1996; Roediger & McDermott, 1995) revealed that prior recall tests inflated both true and false recognition, with the effect increasing with delay (Brainerd et al., 2001). By summarizing data for 14 published studies where recognition tests for DRM lists were either preceded by recall tests or not, Gallo (2006) concluded that prior recall almost always elevated later true recognition, while the effect was weaker and less robust on false recognition. More recently, Brainerd, Bialer et al. (2020a) found that the normed true and false recognition levels following recall tests in Stadler et al. (1999) were only weakly correlated with the levels that were observed when recognition tests were not preceded by recall tests. Moreover, while Roediger et al. (2001) found a negative correlation between true recall and false recognition following prior recall, Brainerd, Bialer et al. (2020a) found that false recognition without prior recall was not correlated with the true recall levels reported in Stadler et al. (1999). In brief, the recognition data in the most commonly used DRM norms are confounded by prior recall tests, and hence, the reported levels of true and false recognition are inflated to unknown degrees. In addition, the reported correlations between recognition and recall are inflated by this same confound. That, in turn, prompted us to conduct new studies to generate recognition norms for the 55 DRM lists that were free of the prior recall confound.

Our studies were also designed to provide norms for a list factor that is a strong predictor of the DRM illusion—gist strength (GS). For background, Roediger et al. (2001) compiled data on a series of list factors that were thought to be potential predictors of the lists’ true and false memory levels. Those factors include backward associative strength (BAS; the probability that list words elicit the critical distractor on a free association test), forward associative strength (FAS; the probability that the critical distractor elicits list words on a free association test), word frequency, word length, word concreteness, and connectivity (the probability that list words elicit each other on a free association test). Roediger et al. conducted multiple linear regressions with these factors and found that (a) BAS was a positive predictor of false recall and false recognition and (b) true recall was a negative predictor of false recall and false recognition. However, as noted before, the recognition results were confounded by prior recall tests.

These findings were consistent with a theoretical account that is known as the activation/monitoring framework (AMF; Roediger et al., 2001). This framework is derived from a combination of spreading activation theory (Anderson & Pirolli, 1984; Collins & Loftus, 1975) and the source monitoring framework (Johnson et al., 1993). AMF assumes that each word is a node in an interconnected associative network, where the “distance” between two nodes depends on the associative strength between the two words (i.e., the probability of a word eliciting another word on a free association task). When a word is encoded, its node will be activated, and such activation will spread to other, connected nodes, with the amount of activation depending on the distance between the two nodes. Therefore, as people encode a DRM list, the critical distractor receives repeated spreading activation from the list words, which stimulates false memories on recall and recognition tests. On such tests, AMF assumes that people attempt to reduce overall levels of false memory by using a source-monitoring operation, which weeds out items that do not retrieve diagnostic details of the encoding phase.

There is another theoretical account of the DRM illusion, fuzzy-trace theory (FTT; Reyna & Brainerd, 1995; Brainerd & Reyna, 1998). FTT posits that people separately store and retrieve two types of episodic representations: verbatim traces of integrated item-specific surface features and gist traces of conceptual content and other elaborative and relational information. Retrieval of verbatim traces stimulates vivid reinstatement of encoded items, which supports true memory and suppresses false memory*.* On the other hand, retrieval of gist traces supports both true and false memory. Because retrieved gist traces are consistent with the conceptual content of encoded items, when people retrieve gist traces, they may determine that they previously encoded both the target items and items that share conceptual content with them. Thus, levels of false memory are directly proportional to the strength of gist retrieval and inversely proportional to the strength of verbatim retrieval. Further, FTT assumes that verbatim and gist retrieval are dissociated, with gist traces fading more slowly than and being more resistant to interference than verbatim traces.

In Roediger et al.’s (2001) study, associative strength—AMF’s main explanatory concept for false memory—was incorporated in the analyses using normed BAS as its quantitative index. However, FTT’s main explanatory concept, GS, was not included in the analyses as a normed quantitative index of GS was not yet available for DRM lists. Thus, comparisons between associative and gist effects are not possible without further norming studies of GS. Another challenge is that associative relatedness and semantic relatedness are often confounded in false memory experiments. In that regard, Brainerd et al. (2008) and Cann et al. (2011) pointed out that words that are associatively related to each other are also semantically related. To illustrate, when Cann et al. (2011) scored the list word-critical distractor pairs of DRM lists for six types of semantic relations, they found that BAS levels were positively correlated with the levels of three types of semantic relations (synonym, category membership, and situational membership). Faced with such challenges, prior studies that attempted to separate gist and associative effects typically relied on proxy gist manipulations while controlling association levels (as indexed by BAS; for a review, see Coane et al., 2021).

**Fig. 1:**
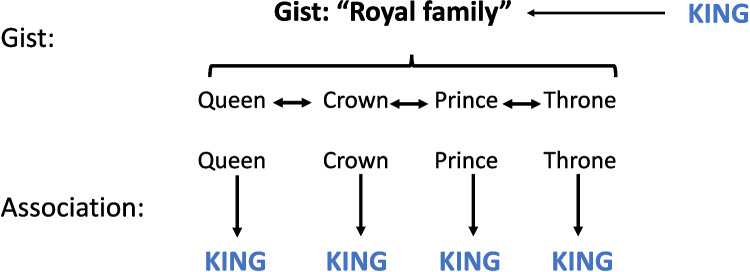
Graphic demonstration of the conceptual and operational difference between gist and association using the *king* list as an example

In sum, as a normed quantitative index of GS has not been available and BAS has often confounded with GS, prior studies of GS and BAS effects on false memory only used indirect, proxy manipulations of GS. To remove these limitations, Brainerd Chang et al. (2020b) developed a quantitative index of GS via a rating task, for a pool of 120 four-word DRM lists, where GS was rated by asking participants to judge the extent to which the words on each list hung together in meaning, on a seven-point numerical scale. In this task, GS is treated as an emergent property of processing the semantic relations that connect list words (see Fig. 1 for a graphic illustration). According to the associative explanation of false memory, each list word sends activation to the critical lure via pre-existing associative links, resulting in high levels of summed activation for the critical distractor. In contrast, the gist explanation posits that as participants encode list words, the gists of DRM lists emerge from processing semantic relations that cut across list words, and critical distractors are likely to be falsely remembered because they fit perfectly with those gists.

Thus, GS is a list-level attribute, and that feature was implemented in the norming procedure used by Brainerd, Chang et al. (2020b). Specifically, participants rated groups of words for their GS—as distinct from the classic procedure of rating individual words for semantic attributes such as valence and concreteness (e.g., Bradley & Lang, 1999; Brysbaert et al., 2014; Warriner et al., 2013). The resulting norms were used to evaluate the GS measure’s reliability and validity. The results showed that GS ratings were both highly reliable (internal reliability estimates >.9) and valid (GS ratings correlated strongly with lists’ objective semantic properties). Importantly, the correlation between mean GS (MGS) and mean BAS (MBAS) for the list pool was .49 (*p* < .001), which is well below the discriminant validity criterion for equivalent measures (*r* = .85). Thus, the null hypothesis that MGS and MBAS are indistinguishable constructs can be rejected. In other words, they each capture information that is not captured by the other. Further, Brainerd, Chang et al. (2020b) demonstrated that MGS and MBAS could be dissociated empirically. Specifically, they manipulated MGS and MBAS factorially in a DRM recognition experiment and found that MGS had significant effects on false recognition of critical distractors regardless of whether MBAS was high or low, such that high-MGS lists always led to higher false recognition than low-MGS lists. However, high-MBAS lists only led to higher false recognition than low-MBAS lists when MGS was low. More recently, Brainerd et al. (2024) reported a parallel series of MGS × MBAS factorial experiments for recall. In standard free recall, they found that only MGS affected false recall while MBAS did not. In forced recall (in which people must recall a fixed number of words) and verbatim + gist recall (in which people recall both encoded words and words that are semantically similar to them), they found that MGS and MBAS had independent effects on false recall. There was also an important MGS-MBAS dissociation for true recall: Increasing MGS improved true recall with all three procedures but increasing MBAS did not.

In sum, a reliable quantitative index of GS is now available that has produced several informative results in recent factorial DRM experiments. Moreover, it has been demonstrated that GS’s effects on true and false memory are different than BAS’s effects (Brainerd, Chang et al., 2020b, Brainerd et al., 2024). Currently, however, GS norms have an important limitation: They are only available for a pool of 120 four-word lists, whereas most DRM studies rely on conventional 15-word lists that produce substantially higher levels of false memory (Robinson & Roediger, 1997; Sugrue & Hayne, 2006). Therefore, GS norms for these longer lists are essential for connecting the MGS versus MBAS comparison with the larger DRM false memory literature.

### The current research

As discussed above, the data for fifty-five 15-word DRM lists in Roediger et al. (2001) provide unconfounded norms for recall but not for recognition. Additionally, prior studies that examined BAS and GS effects have only used indirect proxy manipulations of GS because a quantitative index of GS was not available. In that vein, we conducted three studies that are reported below. Study 1 generated normed recognition data for these 55 lists that were not confounded by prior recall tests. Study 2 generated normed GS ratings for these same lists, using the rating methodology developed by Brainerd, Chang et al. (2020b). In Study 3, the data of the first two studies were combined with the data reported by Roediger et al. (2001), and a series of multiple regression analyses was conducted to parse the contributions of GS, BAS, and other list factors to true and false memory.

## Study 1

### Method

#### Participants and materials

The participants were 530 undergraduate students (*M*_age_ = 19.83, *SD*_age_ = 1.50, 383 females, 255 White, 200 Asian, 32 Black or African American, 28 Hispanic or Latino, 15 prefer not to tell) who participated for course credit. The materials were the fifty-five 15-word DRM lists in the Roediger et al. (2001) norms. We split the lists into three subsets of 19 lists (set A, set B, and set C), with the *king* list appearing in all three sets.^1^ Each participant was administered either set A, B, or C during the study phase. The data collection was conducted over two consecutive semesters. In the first semester, 409 participants were recruited, 135 for set A, 136 for set B, and 138 for set C. This sample size was chosen based on the prior DRM recognition norming study (Stadler et al., 1999), where each list was normed by approximately 110 participants. Given that there were three sets of lists, around 330 were needed, and we chose to oversample to ensure statistical power. However, a programming error caused the critical distractor for the *mountain* list in set B to not be presented during the recognition test. Thus, false memory data for the *mountain* list were missing. While we retained the data of the other 18 error-free DRM lists for these 136 participants, we ran an additional 121 participants for set B with the error corrected to collect false memory data for the *mountain* list. Thus, in the end, there were 135 participants for set A, 257 for set B, and 138 for set C.

#### Procedure

The procedure was closely modeled after Stadler et al. (1999), except that there was no recall test after each list was studied. At the beginning of the study, participants’ informed consent was obtained. Each participant was randomly assigned to set A, B, or C. There were 19 DRM lists in each set, and there were no duplicated words among lists in the same set. The individual lists in each set were encoded and tested over a sequence of three study-buffer-test cycles, with seven lists administered during cycle 1 and six lists administered during cycles 2 and 3. The lists were selected at random with the constraint that there were no duplicated words across lists during a cycle.

During the study phase of each study-buffer-test cycle, participants listened to an audio recording of the lists. The lists were recorded in a neutral voice at a rate of 2 s/word. After each list’s recording was played, a line of asterisks appeared on the computer screen for 5 s to signal the end of the current list, after which the next list appeared. Participants then entered the buffer phase, in which they worked on simple arithmetic problems for 2 min. Following this, the test phase began, and participants were given test instructions. For each test word, participants were asked to judge whether it was an old word that they heard during the study phase or a new word they had not heard. They made the judgment by clicking one of two buttons on the monitor, and the judgment was self-paced. During the first cycle, where seven lists were presented during the study phase, there were 35 test words, including one critical distractor (CD) and three targets (Ts; the list words at the 1st, 8th, and 10 th positions) from each of the seven lists, plus seven unrelated distractors (UDs) that did not resemble any of the list words in meaning or surface form. During the second and third cycles, which contained six lists during the study phase, there were 30 test words, the CD and three Ts from each list, plus six UDs.^2^ There were no duplicated words among the test words across cycles.

### Results

The list-level and trial-level true and false recognition data are available at https://osf.io/qs3fx/. All analyses reported below focused on list-level data. For list-level data, both raw recognition and bias-corrected recognition using the two-high-threshold method (Snodgrass & Corwin, 1988) are provided. Here, raw and bias-corrected recognition were almost perfectly correlated, *r*s = .99 and .96 for false and true recognition, respectively. All the analyses reported in the paper were therefore conducted with raw recognition data, for ease of comparison to Roediger et al. (2001). However, it is worth noting that analyses performed with the bias-corrected recognition data showed the same patterns. We first compared data from the current study to the recognition data of Roediger et al. (2001). Then, we compared the current data with the recognition data from the norming study by Brainerd, Bialer et al. (2020a), which was also not confounded by prior recall. All bivariate correlations were calculated using the *psych* package (Revelle, 2022) in R (R Core Team, 2019).

#### Comparisons between the current study and Roediger et al. (2001)

**Fig. 2:**
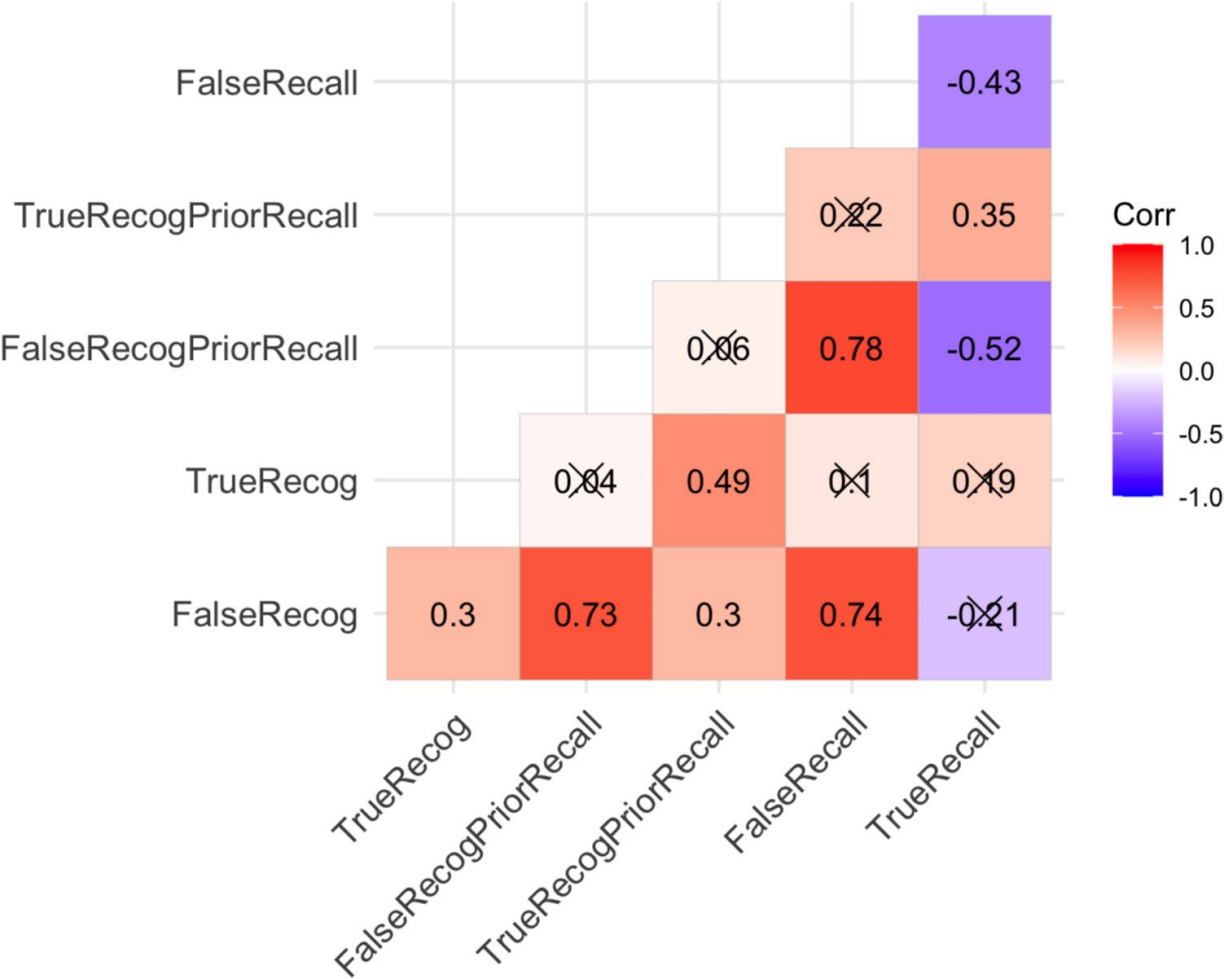
Bivariate correlations between recognition data in the current study and recognition and recall data in Roediger et al. (2001). TrueRecog and FalseRecog are the true and false recognition data collected in the current study. TrueRecogPriorRecall and FalseRecogPriorRecall are the true and false recognition data following prior recall tests that are assembled by Roediger et al. (2001). FalseRecall and TrueRecall are the false and true recall data assembled by Roediger et al. (2001). *Crosses* in the boxes of the plot indicate non-significant correlations

The present recognition data correlated positively with the recognition data preceded by prior recall that were reported by Roediger et al. (2001). The correlation was stronger for false recognition (*r* = .73, *p* < .001), than for true recognition (*r* = .49, *p* < .001), *z* = 2.00,* p* = .045 (see Fig. 2). Consistent with previous findings that prior recall has a more robust effect on true recognition than false recognition (Gallo, 2006), true recognition was significantly higher when it followed prior recall (as in Roediger et al., 2001) than when it did not (as in the current study; *M*s =.80 vs. .66), *t*(108) = 9.99, *d* = 1.87, *p* < .001. False recognition levels remained invariant whether or not they were preceded by recall (*M*s =.59 vs. .60), *t*(108) =.19, *d* = .05, *p* = .847.

Both false recognition with prior recall and without prior recall were strongly correlated with false recall, *r*s =.74 and .78, *p*s <. 001. In contrast, while true recall is positively correlated with true recognition preceded by recall (*r* = .36, *p* = .005), and negatively correlated with false recognition preceded by recall (*r* = –.52, *p* = .002), the true recall data were correlated with neither true recognition nor false recognition when recognition was not preceded by recall. In sum, the results reveal that prior recall affected list-level variability more for true recognition than for false recognition, and it also affected recall–recognition correlations.

#### Comparison between the current study and Brainerd, Bialer et al. (2020a)

Brainerd, Bialer et al. (2020a) collected recognition norms for the 36 DRM lists in Stadler et al. (1999) using the conjoint recognition paradigm (Brainerd et al., 1999, 2022) rather than standard old-new recognition. In conjoint recognition, participants judge the same three types of test words (CDs, Ts, and UDs), but they are assigned to one of three test conditions: (a) Accept Ts and reject both semantically related distractors and UDs (verbatim condition); (b) accept semantically related distractors and reject both Ts and UDs (gist condition); or (c) accept both Ts and semantically related distractors but reject UDs (verbatim + gist condition). The verbatim condition resembles old–new item recognition, but with a minor difference: Participants are explicitly notified of the presence of semantically related distractors on the test.

Given that DRM false memory occurs regardless of whether participants are warned in this manner (McDermott & Roediger, 1998), it is reasonable to expect that the verbatim condition should produce results that resemble our findings for standard old–new recognition. To examine this hypothesis, we computed the bivariate correlation between the recognition data of the 36 Stadler et al. lists in the current study and those of the verbatim condition in Brainerd, Bialer et al. (2020a). As expected, the results showed substantial correlations for both true recognition, *r* = .53, *p* < .001, and false recognition, *r* = .60, *p* < .001.

#### Summary

The normed recognition data in the present study were positively correlated with the corresponding data in two prior recognition norming studies, one preceded by prior recall (Roediger et al., 2001) and the other not preceded by prior recall (Brainerd, Bialer et al., 2020a). More importantly, the present study and the prior literature (e.g., Gallo, 2006) reveal a consistent pattern in which prior recall elevated true recognition, but its effect on false recognition is unreliable.

## Study 2

### Method

#### Participants and materials

The participants were 150 undergraduate students (*M*_age_ = 19.43, *SD*_age_ = 1.14, 101 females, 82 White, 46 Asian, 8 Black or African American, 9 Hispanic or Latino, 5 prefer not to tell) who participated in the study for course credit. The materials were the same fifty-five 15-word DRM lists as in Experiment 1.

#### Procedure

The procedure was similar to that of Brainerd, Chang et al. (2020b; Experiment 1). First, informed consent was obtained. After that, participants read the rating instructions, which stated that they would be presented with a series of 15-word lists, and their task was to rate *how strongly the 15 words* *were related to each other in meaning* using a seven-point scale. Participants were encouraged to use the full scale so that lists for which the 15 words were only weakly related should be given ratings at the low end of the scale (1 or 2); lists for which the 15 words were strongly related should be given ratings at the high end of the scale (6 or 7); and lists for which the 15 words were moderately related should be given ratings in the middle of the seven-point scale (3, 4, or 5). Examples of strongly, weakly, and moderately related lists were constructed and provided to participants to illustrate the rating instructions. An example of a strongly related list would be *university*, *college*, *trade*, *learn*, *homework*, *books*, *test*, *project*, *backpack*, *calculator*, *science*, *math*, *club*, *education*, and *curriculum*; an example of a moderately related list would be *hover, float, plane, wasp, spider, soar, rocket, mosquito, bee, helicopter, baseball, hat, flap, control,* and *space*; and an example of a weakly related list would be *horse, building, relationship, sturdy, comfortable, feed, state, active, distant, monitor, need, cart, jeans, melon, fact,* and *new*.

For each list, the 15 words appeared on the screen one after another in the same order as presented in Roediger et al. (2001), with the next word appearing 2 s after the prior word. This was to encourage participants to process semantic relations among all 15 words rather than basing ratings on a subset of words. When all 15 words were presented and as they remained on the screen, a seven-point rating scale appeared below the list and participants made a self-paced rating.

### Results

The list-level and trial-level data for the GS ratings are available at https://osf.io/qs3fx/, where one can see that there is substantial variability in MGS across the 55 DRM lists (2.04–6.54, *M* = 5.13, *SD* = 1.05). Further, the values of several other list factors that may affect memory, including MBAS, MFAS, connectivity, word frequency, word length, and concreteness, are also included. We report the results in two waves: (a) the reliability and validity of gist ratings and (b) the relation between MGS and MBAS.

#### Reliability and validity of gist strength ratings

As an estimate of the reliability of the normed MGS ratings, we generated 1000 random splits of the participant pool and calculated the Spearman–Brown corrected split-half reliability for each split. This produced an extremely high estimate of internal reliability (*M*_*r*_ =.99, *SD*_*r*_ =.001). This suggests that participants were able to perceive list differences in GS with exceptional precision.

To evaluate validity, we calculated the Pearson bivariate correlations between MGS and (a) distributional semantic similarity and (b) connectivity. Regarding (a), distributed semantic models (DSMs) are computational models that extract high-dimensional vectorized representations of words from large-scale natural language corpora. According to such models, two words’ semantic similarity can be indexed by the distance between their vectors in a multidimensional space, which is determined by statistical regularities in the words’ usage in natural language (Günther et al., 2019). In line with that notion, some prior studies have shown that one of the most commonly used DSMs, word2vec (Mikolov et al., 2013), successfully captures the semantic similarities among DRM list words (Chang & Johns, 2023; Gatti et al., 2022).

To calculate a distributional semantic similarity index, we used a pre-trained word2vec model^3^ to extract word vectors for DRM lists and computed the average frequency-weighted cosine similarity between all possible pairs of list words for each DRM list^4^ (for a similar approach, see Gatti et al., 2022; Marelli & Amenta, 2018). Thus, the distributional semantic similarity indexes were the average levels of semantic similarity among DRM list words. As GS is an emergent property of processing the semantic relations among list words, it should increase as the level of semantic similarity among list words increases. Indeed, we found that the correlation between MGS and distributional semantic similarity was positive and moderately strong, *r* = .53, *p* < .001, supporting the hypothesis that MGS is a valid index of differences in the GS of DRM lists.

Regarding (b), connectivity has been previously used as a proxy measure of GS (e.g., Nelson et al., 2003), and it has been reported that connectivity increases as the density of semantic connections (e.g., synonym, category membership, and situational membership) in a DRM list increases (Brainerd et al., 2008). Here, we found that the correlation between MGS and connectivity was positive and moderately strong, *r* = .50, *p* < .001, again supporting the conclusion that MGS is a valid index of list differences in GS.

#### The relation between MGS and MBAS

MBAS indexes the average associative strength from list words to CDs, which was found to be one of the strongest predictors of false memory by Roediger et al. (2001). However, as pointed out by Brainerd et al. (2008) and Brainerd, Chang et al. (2020b), associative strength and semantic connection are confounded in DRM lists. Therefore, it is reasonable to expect that MGS and MBAS will be positively correlated, and they are. Their correlation was again moderately strong, *r* =.49, *p* <.001. Meanwhile, note that the correlation is far below the discriminant validity criterion for equivalent measures (*r* =.85), which, along with the theoretical and empirical differences discussed above (Brainerd, Chang et al., 2020b, 2024) and the other evidence reported below, supports the conclusion that MGS and MBAS are distinct variables.

## Study 3

Thus far, we have reported normed recognition data that were not confounded by prior recall for the most commonly used DRM list pool (Roediger et al., 2001) in Study 1 and normed GS rating for the same list pool in Study 2. In Study 3, the data of both Study 1 and 2 were combined with the recall and recognition results reported by Roediger et al. (2001), and a series of multiple linear regressions were conducted to examine the effects of BAS and GS on DRM recall and recognition.

### Method

#### Statistical analyses

We first conducted a bivariate correlational analysis of the true and false memory measures and four theoretically motivated variables (MGS, MBAS, MFAS, and connectivity) using the *psych* package (Revelle, 2022) in R (R Core Team, 2019). Next, we used the *lm4* package (Bates et al., 2015) in R to conduct a series of hierarchical linear regressions in order to compare the percentages of variance that MGS and MBAS accounted for. Specifically, we conducted four multiple regressions for each of the following six outcome variables: false recognition and true recognition from the current study, plus false recognition, true recognition, false recall, and true recall from Roediger et al. (2001). We ran three regression models with either MGS, MBAS, or both as predictors, and a fourth model with MGS, MBAS, and the MGS × MBAS interaction as predictors. By comparing the model that includes both MBAS and MGS as predictors (the MBAS + MGS model) to the model that only includes MBAS (the MBAS model) or that only includes MGS (the MGS model), we can control for the shared variance between MBAS and MGS and thus reveal their unique contribution to true and false memory. Such an approach is commonly used to disentangle the effects of related but distinct theoretical constructs (e.g., Adelman et al., 2006; Chang et al., 2023; Chapman & Martin, 2022). Similarly, by comparing the model with MGS, MBAS, and the MGS × MBAS interaction as predictors (the MBAS + MGS + MBAS × MGS model) to the MBAS + MGS model, we can determine the unique contribution of the MGS × MBAS interaction to true and false memory.

In the regression models for false recognition and false recall, we included the same set of covariates as Roediger et al. (2001)—namely, MFAS (Roediger et al., 2001), connectivity (Roediger et al., 2001), frequency of CD (Brysbaert & New, 2009), concreteness of CD (Brysbaert et al., 2014), and level of true recall (Roediger et al., 2001). In the regression models for true recognition, we used the same set of covariates, except that we used mean word frequency and concreteness for list words instead of CDs. In the regression models for true recall, we used the same set of predictors, except that levels of true recall were not included. No multicollinearity problems were detected with any of the regression models as the variance inflation factor (VIF) was always below 2.5. Compared to bivariate correlations, the regression models that include these covariates as predictors can reveal how well MBAS and MGS predict false memory beyond the contributions of other confounding variables. The regression analyses were conducted both with and without a logit transformation of the outcome variables and both sets of analyses produced the same patterns. For simplicity, we only report the regression results without the logit transformation.

### Results

#### Correlational analyses

As shown in Table 1, both MGS and MBAS were positively correlated with false recognition without prior recall (*r*s =.50 vs. .53), and the strength of correlations did not differ reliably, *z* = .21, *p* = .835. Similarly, both MGS and MBAS were positively correlated with false recognition preceded by recall (*r*s = .32 vs. .43), and the strength of correlations was again comparable, *z* = .65, *p* = .513. Meanwhile, the correlation between MBAS and false recall was stronger than that between MGS and false recall (*r*s = .73 vs. .37), *z* = 2.76, *p* = .006. The correlation between true memory and MGS or MBAS is less straightforward. Although neither MGS nor MBAS was correlated with true recall, MGS was positively correlated with true recognition without prior recall, while MBAS was positively correlated with true recognition following prior recall.

**Table 1:** Bivariate correlations between memory measures and memory predictors

	MGS	MBAS	MFAS	Connectivity
False recognition	.50***	.53***	.11	.26
True recognition	.41**	.24	.33*	.47**
False recognition prior recall	.32**	.43**	.12	.03
True recognition prior recall	.25	.36**	.05	.24
False recall	.37**	.73***	.08	–.04
True recall	.04	–.10	.09	.32*

Note. False recognition and true recognition refer to false and true recognition without prior recall that are collected from the current study. False recognition prior recall and true recognition prior recall are false and true recognition data following prior recall tests that are assembled by Roediger et al. (2001). False Recall and True Recall are the false and true recall data assembled by Roediger et al. (2001)

*** <.001, ** <.01, * <.05

For comparison, we also display the correlations for MFAS and connectivity in Table 1. Here, it is especially interesting that although MGS and connectivity are both positively correlated with true recognition without prior recall, only MGS is correlated with false recognition without prior recall. Thus, although MGS and connectivity are positively correlated (*r* = .50, *p* < .001) and conceptually similar, they displayed different relations with false recognition.

#### Regression analyses

##### MBAS effects on true and false memory

We first conducted a series of multiple linear regressions to examine MBAS effects on true and false memory. Similar to Roediger et al. (2001), the regression models included MBAS and the covariates listed above as predictors. As we used word frequency and word concreteness from more recent word norms, our results for those variables could be slightly different than Roediger et al.’s. We first compare our regression results for false recognition with prior recall and false recall to those reported in Roediger et al., and then proceed to other regression models.

The summary of the regressions is presented in Table 2, which includes the model coefficients of all predictors as well as the proportion of variance explained (*R*^*2*^). First, a glance at the third and fifth rows in Table 2 shows that, not surprisingly, we replicated Roediger et al.’s findings that only MBAS and true recall significantly predict false recognition following prior recall and false recall. Then, in the first row, we see that MBAS and true recall also predict false recognition without prior recall, while connectivity is an additional significant predictor.

**Table 2:** Summary of regression results for true and false memory with MBAS and covariates as predictors

	MBAS	MFAS	Connect	Frequency	Length	Concrete	True recall	*R*^*2*^
False recognition	.391**	.088	.292*	.177	.111	.171	– .308*	.421
True recognition	.200	.274*	.342**	.155	.351**	.132	.046	.428
False recognition prior recall	.327*	.152	.160	.150	.086	– .010	– .550***	.474
True recognition prior recall	.394**	– .003	.048	.144	.252	.151	.326*	.339
False recall	.686***	.122	– .020	.018	.088	.103	– .408***	.680
True recall	– .095	– .014	.239	.282	.089	.495**	--	.295

*Notes.* False recognition and true recognition refer to false and true recognition without prior recall that are collected from the current study. False recognition prior recall and true recognition prior recall are false and true recognition following prior recall tests in Roediger et al. (2001). The coefficients for all variables were retrieved from the model that includes MBAS and the covariates as predictors

*** <.001, ** <.01, * <.05

Turning to true recognition without prior recall (the second row in Table 2) and true recognition with prior recall (the fourth row in Table 2), the patterns are less consistent. True recognition with prior recall showed a similar pattern to false recognition with prior recall, as the only significant predictors were MBAS and true recall. For true recognition without prior recall, however, another set of predictors, MFAS, connectivity, and word length, were significant, while neither MBAS nor true recall was significant. Last, the only variable that predicted true recall was word concreteness. Summing up, MBAS’s effects were more robust for false memory than true memory. Moreover, MBAS effects on false recognition are similar with and without prior recall, but they are stronger for true recognition with prior recall than without prior recall.

##### MGS effects on true and false memory

We conducted a similar series of regressions for MGS, where the regression models included MGS and the aforementioned covariates as predictors. The results can be seen in Table 3. In the first, third, and fifth rows, we can see a consistent pattern: MGS, word frequency, and true recall predicted false recognition without prior recall, false recognition with prior recall, and false recall. However, the results for true recognition are again less consistent, as MGS, MFAS, and word length predicted true recognition without prior recall (the second row in Table 3), but no variables predicted true recognition with prior recall (the fourth row in Table 3). Last, the only significant predictor of true recall was again word concreteness. Therefore, similar to MBAS, MGS’s effects were more robust with false memory than true memory and are consistent across false recognition with and without prior recall. In contrast to MBAS, MGS’s effects were stronger for true recognition without prior recall than with prior recall, which echoes the bivariate correlation results.

**Table 3:** Summary of regression results for true and false memory with MGS and covariates as predictors

	MGS	MFAS	Connect	Frequency	Length	Concrete	True recall	*R*^*2*^
False recognition	.456**	.158	.107	.353**	.081	.178	– .273*	.452
True recognition	.323*	.327**	.193	.182	.323*	.131	.054	.453
False recognition prior recall	.403**	.215	– .007	.298*	.063	– .010	– .518***	.505
True recognition prior recall	.171	.052	– .001	.034	.244	.152	.300	.233
False recall	.431**	.160	– .138	.320**	– .001	.218	– .403**	.502
True recall	– .113	– .035	.289	.283	.098	.495**	--	.295

*Notes.* False recognition and true recognition refer to false and true recognition without prior recall that are collected from the current study. False recognition prior recall and true recognition prior recall are false and true recognition following prior recall tests in Roediger et al. (2001). The coefficients for all variables were retrieved from the model that includes MGS and the covariates as predictors.

*** <.001, ** <.01, * <.05

##### Comparison of MGS and MBAS effects

As shown in Tables 2 and 3, MGS and MBAS exhibited similar qualitative patterns in predicting false memory but different patterns in predicting true memory. To parse their contributions, we conducted a series of regressions that included both MGS and MBAS as predictors, with all other covariates, too. We focus on the regression models for false recognition, true recognition, and false recall, as the previous results showed that neither MGS nor MBAS predicted true recall.

In the first and second columns of Table 4, we report the model coefficients for MGS and MBAS when they are both included in the regression models as predictors. We focus first on false memory models. There, we see that MGS predicted false recognition, with or without prior recall, while MBAS did not. In contrast, MBAS predicted false recall, while MGS did not. Thus, MGS’s effects on false recognition are more robust than MBAS’s effects, whereas the opposite is true for false recall. Turning to true recognition, for the regression model without prior recall, neither MGS nor MBAS was a reliable predictor. However, for the regression model with prior recall, MBAS was a reliable predictor.

**Table 4:** Model coefficients for and unique variance explained by MGS, MBAS, and their interactions

	Model coefficients			Unique Δ*R*^*2*^ explained (in %)		
	MGS	MBAS	Interaction	MGS	MBAS	Interaction
False recognition	.343*	.218	–.337*	**10.920**	4.553	11.607
True recognition	.271	.109	.122	**7.296**	1.695	1.645
False recognition prior recall	.316*	.168	–.285*	**8.486**	2.469	7.938
True recognition prior recall	–.022	.401**	–.013	.063	**31.229**	.025
False recall	.100	.636***	–.229*	.645	**26.731**	4.042
True recall	–.080	–.067	.251	1.003	1.010	10.286

Notes. False recognition and true recognition refer to false and true recognition without prior recall that are collected from the current study. False recognition prior recall and true recognition prior recall are false and true recognition following prior recall tests in Roediger et al. (2001). The coefficients for MGS and MBAS were retrieved from the model that includes MGS, MBAS, and the covariates as predictors. The coefficients for the MGS × MBAS interaction were retrieved from the model that includes MGS, MBAS, the covariates, and the MGS × MBAS interaction as predictors. Δ*R*^*2*^ (in %) refers to the unique variance accounted for by the given variable relative to the other variable. The larger Δ*R*^*2*^ explained (in %) in the MGS versus MBAS comparison is present in bold face

*** <.001, ** <.01, * <.05

In the fourth and fifth columns of Table 4, we report the unique Δ*R*^*2*^ accounted for by MGS versus MBAS. This was calculated by comparing the model that included both MGS and MBAS to the model that included either MGS or MBAS. To illustrate, the unique Δ*R*^*2*^ in false recognition explained by MGS is calculated by dividing the Δ*R*^*2*^ between the model that only included MBAS and the model that included both MGS and MBAS by the total *R*^*2*^ of the latter model. Thus, the unique Δ*R*^*2*^ in false recognition that is accounted for by MGS indexes the unique contribution of MGS to false recognition beyond that of MBAS. We see that for both true and false recognition without prior recall, MGS accounted for more unique variance than MBAS. Additionally, MGS accounted for more unique variance in false recognition with prior recall than MBAS, while MBAS accounted for more unique variance in true recognition with prior recall than MGS. Last, as for false recall (in the fifth row of Table 4), MBAS accounted for more unique variance than MGS did. Again, a pattern emerges that is consistent with prior results—namely, that MGS is a stronger predictor of false recognition while MBAS is a stronger predictor of false recall. In addition, MGS is a better predictor of true recognition without prior recall, while MBAS is a better predictor of true recognition with prior recall.

##### Interactions between MGS and MBAS

**Fig. 3:**
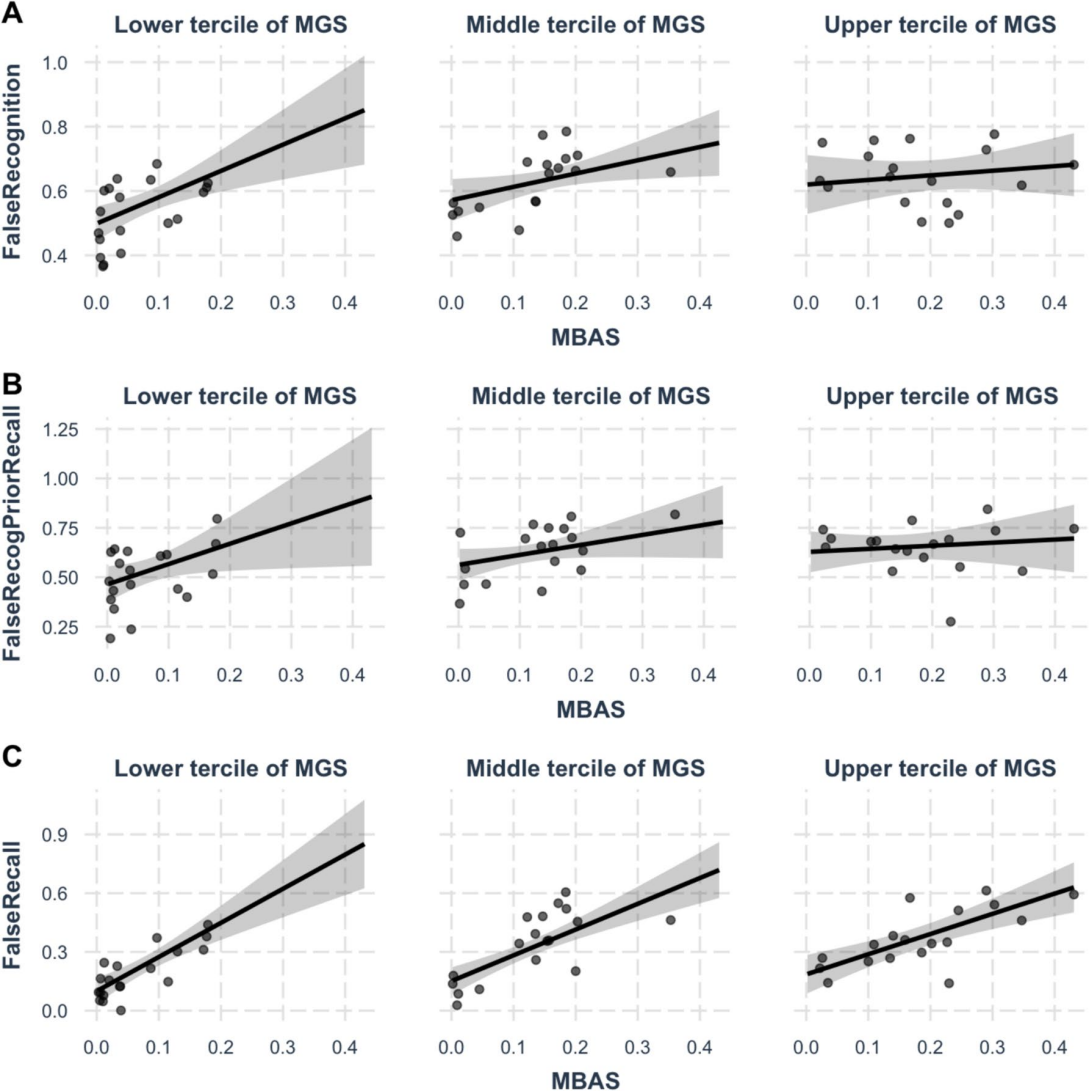
MBAS’s effects on **A** false recognition without prior recall, **B** false recognition preceded by prior recall, and **C** false recall across upper, middle, and lower thirds of the distribution of MGS

To investigate MGS × MBAS interactions, we ran a final series of regression models that included MGS, MBAS, and their interaction as predictors. In the third and sixth columns of Table 4, we report the model coefficients and unique Δ*R*^*2*^ explained by the MGS × MBAS interaction. The unique Δ*R*^*2*^ was calculated in a similar way as indicated above, by comparing the model that included MGS, MBAS, and their interactions to the model that only included MGS and MBAS. There was a consistent pattern that for all false memory measures (false recognition without prior recall, false recognition with prior recall, and false recall), the coefficient for the MGS × MBAS interaction was significant and negative. To facilitate the interpretation of this result, simple slope analyses and interaction plots for the MGS × MBAS interaction in the three false memory regression models are shown in Table 5 and Fig. 3. The table and plots reveal that MBAS’s effects shrink as MGS increases, which again suggests that MGS and MBAS have distinct effects on false memory.

**Table 5:** Results of the simple slope analysis for the interactions between MBAS and MGS

	Regression coefficients for MBAS		
	MGS = 4.29 (Lower tercile median)	MGS = 5.37 (Middle tercile median)	MGS = 6.09 (Upper tercile median)
False recognition	.82*	.41*	.14
False recognition prior recall	1.03*	.51	.16
False recall	1.74***	1.32***	1.03***

*Note*. Model coefficients for MBAS are shown in the table

*** <.001, ** <.01, * <.05

Additionally, the Akaike information criterion (AIC) is reported for all aforementioned regression models in Table 6.^5^ In that connection, a lower AIC indicates a better model fit, and a ΔAIC > 2 was generally considered substantial evidence in favor of the model with a lower AIC (Burnham & Anderson, 2004). As seen in Table 6, for false recognition (both with and without prior recall) and false recall, the model that includes MGS, MBAS, and their interaction provided the best model fit. For true recognition without prior recall, the MGS model provided the best fit, whereas for true recognition with prior recall, the MBAS model provided the best fit.

**Table 6:** Akaike information criterion (AIC) of regression models

	AIC			
Models	MGS	MBAS	MGS +MBAS	MGS +MBAS +MGS×MBAS
False recognition	140.04	142.97	139.83	**134.93**
True recognition	**139.85**	142.39	141.06	142.27
False recognition prior recall	134.41	137.76	134.97	**131.63**
True recognition prior recall	158.46	**150.40**	152.28	154.28
False recall	134.77	110.37	111.60	**108.33**
True recall	151.86	151.86	153.63	152.89

*Notes.* Covariates are included in all regression models. MGS refers to the model that includes MGS and covariates as predictors. MBAS refers to the model that includes MBAS and covariates as predictors. MGS + MBAS refers to the model that includes MGS, MBAS and covariates as predictors. MGS + MBAS +MGS × MBAS refers to the model that includes MGS, MBAS, their interaction, and covariates as predictors. False recognition and true recognition refer to false and true recognition without prior recall that are collected from the current study. False recognition prior recall and true recognition prior recall are false and true recognition following prior recall tests in Roediger et al. (2001). The lowest AIC among the four models is highlighted in bold fonts

## General discussion

In this article, we first reported two norming studies of the most commonly used pool of 55 DRM lists (Roediger et al., 2001)––specifically, a recognition study (Study 1) and a GS rating study (Study 2). Study 1 provided normed DRM recognition data unconfounded by prior recall, which were positively correlated with the recognition data in the two prior recognition norms (Brainerd, Bialer et al., 2020a; Roediger et al., 2001). Here, the correlation was stronger for false recognition than for true recognition, and only true recognition was significantly higher when preceded by recall than when not preceded by recall, which echoes the extant literature (Gallo, 2006). Study 2 provided norming data for the GS index of DRM lists, which showed that the GS index is highly reliable and valid. Next, in Study 3, we combined the two studies’ data with data for the same list pool assembled by Roediger et al. (2001) and compared the effects of gist versus associative strength on true and false memory. Below, we discuss the implications for the comparison of BAS and GS and their interaction in the DRM paradigm.

### Gist versus associative strength in false memory

A rating procedure similar to that used by Brainerd, Chang et al. (2020b) was implemented to collect MGS values for the 55 DRM lists. These normed values were both a reliable and valid index, and hence, they supplied our operational definition of GS. Previously, the lack of such an index precluded measurement of inter-list differences in GS, which makes it difficult to use GS to explain inter-list differences in false memory. For instance, it was uncertain whether GS differs between the list whose CD is *bitter*, which produces low levels of false memory, and the list whose CD is *sweet*, which produces high levels of false memory. We now know that the answer is yes as MGS is 5.55 for the *sweet* list and 3.75 for the *bitter* list, which means that people perceived stronger meaning connections among the words on the first list than among the words on the second.

Considering that the commonly used index of associative strength (MBAS) has been widely discussed in the false memory literature, it is important to understand the relation between MGS and MBAS and compare their contributions to false memory. Our results showed that MGS and MBAS are positively correlated (*r* =.49), which makes sense as words that are associated with a CD also tend to share semantic connections. However, the correlation is far from perfect, meaning that MGS and MBAS are distinguishable. This distinguishability was further illustrated by a series of multiple regressions that compare MBAS’s and MGS’s contributions to false memory (Tables 2, 3, and 4). Table 4 provides a picture of the comparative effects of MBAS and MGS on false memory. While MBAS is a stronger predictor of immediate false recall, MGS is a stronger predictor of false recognition. This difference is conceptually consistent with prior findings. According to Roediger et al. (2001), MBAS is a stronger predictor of false recall than false recognition (*r*s =.73 vs..41). When Brainerd, Chang et al. (2020b) and Brainerd et al. (2024) factorially manipulated MBAS and MGS in recognition and recall experiments, they also reported that MBAS’s effects were more robust with false recall. Specifically, they found that higher MBAS only increased false recognition when MGS was low. In contrast, higher MBAS increased false recall regardless of whether MGS was high or low, although this effect was restricted to forced recall. In free recall, only higher MGS increased false recall while MBAS had no effect.

Furthermore, Brainerd, Chang et al. (2020b) showed that GS had stronger effects on false recognition than BAS, as higher MGS increased false recognition regardless of whether MBAS was high or low. Using a 15-word DRM list pool that is more representative of the word lists used in the broader false memory literature, we found similar evidence for a more robust effect of MGS than MBAS, regardless of whether recognition tests were preceded by recall or not (see Table 4). However, we did not observe as strong an effect for MGS in false recall as Brainerd et al. (2024) did. In our analyses, the MGS effect on false recall was weaker than the MBAS effect. A possible reason for the discrepancy is a key difference in the recall tests used between Roediger et al. (2001) and Brainerd et al. (2024). The recall data reported in Roediger et al. (2001) were collected with an immediate recall test after each 15-word list was presented. In contrast, the latter study administered a final recall test after eight four-word lists (i.e., a total of 32 words) were presented. This has been a more commonly used method in DRM research since Payne et al. (1996), Toglia et al. (1999), and other early DRM studies. According to FTT, the effect of longer lists reduces verbatim retrieval relative to immediate recall of shorter lists. Therefore, it is possible that the effects of MGS were stronger in Brainerd et al. (2024) because the procedure forced participants to rely more on gist retrieval.

In summary, MGS was a consistently stronger predictor of false recognition than MBAS. While MBAS’s effects were more pronounced than MGS’s in immediate false recall, its effects may be comparable to or weaker than MGS’s effects when participants recall longer lists (Brainerd et al., 2024). These findings are in agreement with some prior arguments that different mechanisms contribute to memory in different test formats (Gallo & Roediger, 2002). Moreover, the fact that MBAS’s effects are strongest in immediate recall following single-list presentation is congruent with the previous finding that associative activation is relatively transient. For example, Meade et al. (2007) found an associative priming effect for CDs in a lexical decision task only when the test was administered within a few seconds of list presentation. Similarly, Tse and Neely (2005) reported that associative priming effects for DRM lists only remain detectable for about 35 s.

### Interactions between gist and associative strength in false memory

In the regression models for the three false memory measures (false recognition without prior recall, false recognition preceded by recall, and false recall), there was a consistent interaction between MGS and MBAS (see Table 4). The coefficients for the interactions were always negative, indicating that the MBAS effect becomes weaker as MGS increases (see Table 5 and Fig. 3). This parallels the findings of Brainerd, Chang et al. (2020b). When those authors used a 2(MGS: high, low) × 2(MBAS: high, low) factorial design, they found an MGS × MBAS interaction in false recognition, which revealed that MBAS had significant effects on false recognition when MGS is low but not when MGS is high.

This interaction is informative for both FTT and AMF as well as the false memory literature in general. The fact that MGS moderated MBAS’s effects again provides strong evidence that MGS and MBAS are distinct variables when it comes to their influence on false memory. The interaction also suggests that gist and associative strength can be competing processes, as their effects appear to trade-off. A direct empirical implication is that MGS and MBAS should both be considered when researchers attempt to select DRM lists that will elicit high versus low levels of false memory. It is not sufficient to just pick lists with high versus low MBAS, as the differences in false memory levels between high-MBAS and low-MBAS lists would depend on the MGS levels of those lists. Specifically, the differences would be larger when gist is weak than when gist is strong.

Why do MBAS’s effects wane as MGS increases? One hypothesis is that BAS and GS compete for attention during encoding. More explicitly, when people study a strong-gist list, they may focus on how each word is related to the other words at the expense of words that come to mind via associative connections. To illustrate, when studying a strong-gist list such as the *army* list (*navy, soldier, United States, rifle, air force, draft, military, marines, march, infantry, captain, war, uniform, pilot, combat*; MGS = 6.53, MBAS =.14), where all the words have military meanings, participants may focus on processing the salient military content rather than associates of list words. In this case, semantic processing may even suppress associative processing. However, for a relatively weak-gist list such as the *black* list (*white, dark, cat, charred, night, funeral, color, grief, blue, death, ink, bottom, coal, brown, gray*; MGS = 3.39, MBAS =.13), where words cannot be easily grouped according to a common gist, attention to the inter-word relations may to some extent be shifted towards processing associates of individual words. Meanwhile, one should not neglect that the MGS × MBAS interaction can also be interpreted as MBAS moderating the MGS effect. By similar logic, high-MBAS lists may draw participants’ attention from processing inter-word relations to associates of individual words, thus weakening the MGS effect relative to weak-associative lists. Although the current study was not designed to test these hypotheses, the interaction between MGS and MBAS could be a promising research question for future studies to explore.

### Additional considerations and limitations

The BAS indexes for DRM lists used in the present study and Roediger et al. (2001) were calculated based on the word association norms of Nelson et al. (2004), which is the standard in the literature. Those norms were collected about 20 years earlier than the MGS ratings in the present study. Here, one may question whether the comparison between MBAS and MGS is a fair one, given the difference in the time of data collection. There may be generational differences in the perception of associative strength for the DRM lists that were not captured by the BAS indexes generated 20 years ago. Thus, MBAS might be a stronger predictor if more recent association norms were used. To test this possibility, we calculated a new MBAS index for the 55 DRM lists using more recent free association norms (De Deyne et al., 2019) and reran the regression models. The regression results are shown in Table 7, where it can be seen at a glance that the patterns are the same as those reported in Table 4. Therefore, we conclude that the key findings reported here are robust.

**Table 7:** Model coefficients for and unique variance explained by MGS, MBAS, and their interactions with the MBAS derived from new free association norms

	Model coefficients			Unique Δ*R*^*2*^ explained (in %)		
	MGS	MBAS	Interaction	MGS	MBAS	Interaction
False recognition	.424**	.071	–.431**	**19.06**	.58	14.81
True recognition	.259	.147	.035	**6.82**	3.29	.10
False recognition prior recall	.362*	.092	–.335*	**12.37**	.87	8.57
True recognition prior recall	.029	.326*	–.206	.13	**24.39**	5.27
False recall	.245	.411**	–.286*	4.91	**14.77**	5.58
True recall	–.073	–.090	.137	.86	1.92	2.50

Notes. False recognition and true recognition refer to false and true recognition without prior recall that are collected from the current study. False recognition prior recall and true recognition prior recall are false and true recognition following prior recall tests in Roediger et al. (2001). The MBAS index was derived using the free association norms of De Deyne et al. (2019). The coefficients for MGS and MBAS were retrieved from the model that includes MGS, MBAS, and the covariates as predictors. The coefficients for the MGS × MBAS interaction were retrieved from the model that includes MGS, MBAS, the covariates, and the MGS × MBAS interaction as predictors. Δ*R*^*2*^ (in %) refers to the unique variance accounted for by the given variable relative to the other variable. The larger Δ*R*^*2*^ Explained (in %) in the MGS versus MBAS comparison is present in bold face.

*** <.001, ** <.01, * <.05

A similar time differential applied to the normed recognition data, with a 20-year gap between the current study and Roediger et al. (2001). Despite the fact that we used the same materials and a similar undergraduate participant pool, and we closely modeled the procedure of the prior study, we cannot rule out possible generational differences in true and false recognition. However, we do not think the time differential is a serious threat to the validity of our findings for the following reasons. First, our key findings are highly consistent with the prior false memory literature. For instance, the finding that prior recall elevated subsequent true recognition more than false recognition aligns with abundant previous evidence (see Brainerd & Poole, 1997; Gallo, 2006 for reviews). Similarly, the finding that MGS is a stronger predictor of false recognition than MBAS agrees with the prior findings of Brainerd, Chang et al. (2020b) and Brainerd et al. (2024). Second, even if there are systematic generational differences in false memory, such differences are unlikely to favor MBAS over MGS or vice versa, which is of primary interest in the present study. As evidence for that, the correlations between MGS and false recognition are not significantly different across the two datasets collected more than 20 years apart, *z* = 1.11, *p* = 0.267, nor are the correlations between MBAS and false recognition, *z* =.66, *p* = 0.507. Moreover, the key patterns are replicated in data from both the current study and Roediger et al. (2001), such that MGS is a stronger predictor of false recognition than MBAS, and there is an interaction between MGS and MBAS in false memory. This suggests that the observed patterns are quite stable and not unduly influenced by the time differential.

Another potential limitation lies in the fact that both the MBAS and MGS indexes used in this study are derived from human behavioral tasks, namely the free association task for MBAS and gist ratings for MGS. In that regard, it has been discussed in the literature that using human data to explain human data can lead to concerns (Jones et al., 2015; Westbury, 2016). For example, given that the free association task shares a common retrieval process with free recall, it is possible that the task similarity contributes to the result that MBAS is a better predictor of false recall beyond the theoretical difference. In that connection, the current study adopted a common method of operationalizing the theoretical latent variables (associative strength and gist strength) with quantitative indexes (MBAS and MGS) that are constructed based on human data. However, alternative approaches also exist, which offer alternative gist and associative strength indexes that are independent of human annotation (e.g., Fradkin & Eldar, 2023). Therefore, future research is recommended to explore the alternative approximations of associative and gist strength.

## Conclusion

The present study adds important normative data on the most commonly used false memory list pool, in the form of recognition data unconfounded by prior recall and GS ratings. We used these data to compare the effects of associative strength (indexed by MBAS) and gist strength (indexed by MGS) on false memory in a series of multiple regressions. We concluded that recognition and the recall–recognition relation are modified by prior recall, and such effects were stronger for true recognition than for false recognition. Moreover, MGS had stronger effects on false recognition, regardless of whether prior recall tests were administered, while MBAS had stronger effects on immediate false recall. Last, there was a consistent interaction between MGS and MBAS in false memory, suggesting that MBAS’s influence weakens as MGS increases. These findings also provide critical tests of the two major theories of the DRM illusion.

## Data Availability

All data, materials, and codes are available at https://osf.io/qs3fx/.

## Footnotes

1 Thus, the data reported for the *King* list were the average values over the three sets.

2 Due to a programming error, the data for one T for the *bread*, *carpet*, *high*, *lamp*, and *stove* lists in set A and for the *king* list in set C had to be removed because those Ts (*dough*, *bag*, *cliff*, *lighter*, *lid*, and *rule*, respectively) were presented both as Ts and UDs on the test. Thus, true recognition for those five lists is based on two Ts rather than three. Similar errors occurred for the *cold*, *king*, *citizen*, *music*, and *thief* lists in set B during the first semester, and those errors were corrected during the second semester. Thus, true recognition for those five lists is based on two Ts for first semester participants and three Ts for second semester participants.

3 The model is available at https://code.google.com/archive/p/word2vec/.

4 Four DRM lists whose critical distractors are *army*, *citizen*, *city*, and *swift* are excluded because some list words are not available in word2vec. Thus, a total of 51 lists were included in the analysis. Word frequency is obtained from the SUBLEX word frequency norm (Brysbaert & New, 2009).

5 We thank two reviewers of the manuscript for the recommendation of reporting AIC here.
